# Supplementary material for: Acceptance and compliance with micronutrient powder (MNP) among children aged 6–23 months in northern Nigeria
Source: PLOS Glob Public Health. 2022 Oct 17;2(10):e0000961. doi: 10.1371/journal.pgph.0000961 (PMC10022258; doi:10.1371/journal.pgph.0000961)
Supplement: S1 Dataset — (ZIP) [file pgph.0000961.s008.zip › S8_Dataset/In-depth Interviews.zip!/In-depth Interviews/110715_K_DW_TA_2_CG.b.pdf]

PHASE TWO (2) FEEDING TRIAL IN-DEPTH INTERVIEW WITH A FEMALE CAREGIVER AT DIDOGO, DANKO/WASAGU LOCAL GOVERNMENT AREA OF KEBBI STATE
CONDUCTED BY AMINA DUNIYA AND TRANSCRIBED BY TIMOTHY AUTA ON 11TH JULY, 2015

I: Thank you for the opportunity to discuss with you. Could you please tell me how many you are in the family?

R: The young girls are three and three boys.

I: What about the adults?

R: We are two.

I: Who and who?

R: Myself and my husband

I: Thank you. Among your children, how many among them are between 6-23 months?

R: Honestly, no one is six months; only this one that is almost two years.

I: Like her now, what type of food does she eat?

R: She use to eat tuwo, fura, shinkafa and dame, if the squash it for her.

I: What is dame?

R: Dame is tuwo squashed in water to look like gruel or fura.

I: Thank you. We have heard of food challenges for young children in this setting related to the seasons. What ways can these food challenges are coped?

R: We use to get food here always, if there is health.

I: Ok. Thank you. One thing we heard in other interviews that caregivers often give their children 6-23 months "tea". Could you please define or explain "tea"?

R: (She laughed).

I: What is "tea"?

R: What is "tea"?

I: Yes.

R: Tea is prepared with hot water, milk and other beverages.

I: Thank you. Now, I want us to talk about the MNP that you have received. Could you tell me about your experience with the product?

R: Honestly, it has made her to have good body...

I: (Sielnce...)

R: It has made her to have good body. When I give it to her, she doesn't take it and sometimes vomits it. If I put it for her in rice or soup, she eats it.

I: So you mix it for her in the rice or soup?

R: Yes.

I: Only these two?

R: No. I also put it inside kunu. But when I put it inside kunu, she doesn't take.

I: Thank you. But since you started giving her the product, have you noticed any changes like increase in food consumption or any other thing?

R: Yes. Before she used to eat, but she now eats a lot and she has added weight. But at the moment, she doesn't eat again and her body is not as good as it used to be.

I: When did you start observing this?

R: Almost two weeks now since she stopped eating well.

I: What do you think made her to develop good body before?

R: How will I know?

I: From your perspective.

R: Because she was eating well.

I: Thank you. Some caregivers mentioned that it was hard to store the MNP without it being taken by others or eaten by rodents. What was your experience?

R: No. I don't have such experience. I didn't keep it where others will see and take it. When other children say they want to take, I do deny them. When the baby is taking, the other boy will fetch and run away. He will fetch and start running. What will I do when he has put it in his mouth?

I: Ok. Thank you. What do you perceived to be side effects of this MNP?

R: Honestly, I have not seen any side effect.

I: Thank you. Some mothers indicated that it might be hard to remember to use MNP. What was your experience?

R: That woman told us that if we don't remember to give the baby, we can give her twice the next day. That was what she said. If we forget to give in a day, we can give two the next day.

I: For you now, was there a day you forgot to give?

R: Honestly, i forgot.

I: When you forgot, what happened the next day?

R: When I forgot, I gave her two the next day.

I: Thank you. What specific solutions can you suggest to address each of the above mentioned barrier or challenge?

R: (She laughed). I don't have any suggestion. Anyone that forget should try to remember. (She keeps laughing).

I: Thank you. When do you usually give the MNP to the child?

R: Honestly, sometimes in the morning or afternoon. If I give her in the morning and she refused to take, I do leave it till afternoon.

I: OK. Thank you. You earlier mentioned increase in food consumption when you started giving her the MNP. Like what foods did she consumed more?

R: It is the tuwo.

I: What about breast milk?

R: I have weaned her.

I: Thanks. In other communities, we noticed that sharing of the MNP occurred. Could you talk any sharing of the MNP when you used it?

R: If that is done, will it still benefit the index child?

I: Did you do it?

R: No. I didn't do that.

I: If you gave her and there is leftover, what do you do?

R: I pour it away.

I: Thank you. Will the 60 sachets be enough for the 60 days?

R: Honestly, I sometimes give the child two. It won't be enough.

I: Thanks so much. Do you see the MNP as a food, a medicine or something else?

R: It is not a medicine, since the woman said it is not medicine.

I: In your perception what do you see it as?

R: I see it as a medicine.

I: Why do you say it is a medicine?

R: It is something I was given and it is related to health. Anything that has to do with health, to me is a medicine.

I: Tell me whether you would purchase this product if it were cheaply on sale in your community?

R: Yes, I will pay for it.

I: What will make you buy it?

R: Because it is something that is good for the health of my child, I will purchase it.

I: How much would you be willing to pay for a sachet?

R: I will be willing to pay six hundred naira (600) only.

I: Thank you so much. What are the best community channels to distribute the product?

R: It should be brought to the community leader's place and through Alhaji

I: Who is Alhaji in this community?

R: (She laughed) ... It should just be brought and given to him.

I: OK. Thank you. Who influenced your decision regarding feeding your baby the MNP?

R: My husband.

I: In this community, who do you trust the most for receiving messages about infant and young child feeding?

R: In this community, no one trusts the other. We are all living on our own.

I: OK. Thank you. Now please tell me some ways that you believe would be effective for promoting MNP to caregivers in the community so that they would comply with using it appropriately?

R: It should be through Mallam Ibrahim.

I: Who is Mallam Ibrahim?

R: He is educated in this community. We all know him to be enlighthen.

I: Thank you so much. You have mentioned community leader and Mallam Ibrahim, are there other ways?

R: There is no other way.

I: Thank you. How would you explain to other community members how to use it?

R: I would tell them one per day.

I: What again?

R: I would tell them to mix it with kunu, squashed tuwo, rice or soup for children to eat.

I: If there is leftover, what happens?

R: It should not be given to older child, only meant for the index child.

I: If the child doesn't finish it, what happens to the leftover?

R: One should pour it away.

I: Why won't you give the leftover to other children?

R: It was said they should not be given. Since, it has been said they should not be given, will one give them?

I: Ok. Thank you. Is there any message or recommendation to develop an effective program?

R: No, I don't have any recommendation.

I: Thank you so much.
